# Highlight report: Imaging of bile ducts and the bile canalicular network

**DOI:** 10.17179/excli2019-1548

**Published:** 2019-07-04

**Authors:** Regina Stoeber

**Affiliations:** 1 Leibniz Research Centre for Working Environment and Human Factors

## ⁯⁯⁯

Cholestasis is defined as reduced bile flux, either caused by an obstruction of bile ducts or by compromised hepatocellular secretion (Pollheimer et al., 2014[13]; Fickert et al., 2014[3]; Slitt et al., 2007[17]; Jansen et al., 2017[11]). In the acute phase of obstructive cholestasis ruptures of the apical (bile canaliculi forming) hepatocyte membranes occur that lead to bile salt rich necrotic regions, the so-called bile infarcts (Ghallab et al., 2019[5]). In the chronic phase, the biliary tree undergoes massive remodeling by branching of interlobular ducts and re-looping so that an initially sparse network around portal veins transforms into a much denser network (Vartak et al., 2016[20]). For a better understanding of the complex architectural changes it is necessary to 3D-reconstruct and quantify the changes during cholestasis. For this purpose, Damle-Vartak and colleagues recently published a protocol to visualize the finest domains of the biliary network, bile canaliculi and interlobular ducts, based on immunofluorescence, 3D-confocal imaging, surface reconstruction and quantitative morphometry (Damle-Vartak et al., 2019[1]). The method is applicable to all types of liver injury and can be used for cleared tissue of approximately 500 µm thick sections. 

One important discovery made by this method is that the most upstream domain of the biliary tract, bile canaliculi, responds differently to cholestasis than the more downstream interlobular ducts (Damle-Vartak et al., 2019[1]). Bile canaliculi adopt wider diameters and form spine-like protrusions; while the ducts undergo elongation, branching and looping to form a denser mesh without increasing their diameters. 

Recently, much effort has been invested to gain a better understanding of cholestasis (Ehrlich and Glaser, 2018[2]; Pradhan-Sundd et al., 2018[14]; Sultan et al., 2018[18]; Thompson et al., 2018[19]) and associated hepatotoxicity (Sezgin et al., 2018[16]; Leist et al., 2017[12]; Grinberg et al., 2014[7]; Godoy et al., 2013[6]). Often, these studies require quantitative image analysis as a precondition for mathematical modeling (Hoehme et al., 2010[9], 2017[10]; Hammad et al., 2014[8]; Schliess et al., 2014[15]; Ghallab et al., 2016[4]). The just published protocols (Damle-Vartak et al., 2019[1]) offer excellent preconditions to study the spatial responses of the biliary tree and can in principle be applied to analyze any 3D-conduit-like structure. 

## Conflict of interest

The author declares no conflict of interest.
